# Human urinary kallindinogenase therapy for acute ischemic stroke according to Chinese ischemic stroke subclassification: Clinical efficacy and risk factors

**DOI:** 10.1002/brb3.1461

**Published:** 2019-12-02

**Authors:** Si‐Qia Chen, Dong‐Yang Mao, Dun‐Can Wei, Wen‐Zhen He

**Affiliations:** ^1^ Department of Neurology First Affiliated Hospital of Shantou University Medical College Shantou China; ^2^ Clinical Pharmacy First Affiliated Hospital of Shantou University Medical College Shantou China; ^3^ Department of Pharmacy First Affiliated Hospital of Shantou University Medical College Shantou China

**Keywords:** acute ischemic stroke, Chinese ischemic stroke subclassification, homocysteine, human urinary kallidinogenase, large artery atherosclerosis, risk factor

## Abstract

**Introduction:**

To evaluate effectiveness of human urinary kallindinogenase (HUK) in patients with acute ischemic stroke (AIS) according to Chinese ischemic stroke subclassification (CISS) and analyzed risk factors of clinical efficacy.

**Methods:**

In this retrospective study, 134 patients received conventional therapy were enrolled to control group, and 132 patients received HUK treatment were enrolled to HUK group. National Institute of Health Stroke Scale (NIHSS) score was used to evaluate the clinical efficacy. Multivariate analysis of risk factors was performed by using logistic regression.

**Results:**

After treatment, NIHSS score of HUK group was significant lower than that of control group (*p* = .009). Effectiveness rate was 71.2% in HUK group, and 53.7% in control group, respectively (*p* = .003). The NIHSS of patients with large artery atherosclerosis (LAA) subtype in HUK group was significantly lower than that in control group (*p* = .005). The absence of HUK (OR = 2.75), homocysteine (OR = 0.15), and CS subtype (OR = 0.18) were risk factors for HUK clinical efficacy.

**Conclusions:**

Human urinary kallindinogenase is an effective therapeutic approach for treatment of patients with AIS, especially in patients with LAA subtype. The absence of HUK, elevated homocysteine, and cardiogenic stroke subtype were risk factor for clinical efficacy of HUK.

## Graphical abstract

In this study, we aimed to evaluate the effectiveness of human urinary kallindinogenase (HUK) in patients with acute ischemic stroke (AIS) according to Chinese ischemic stroke subclassification and analyzed the risk factors of the clinical efficacy. We found that HUK is an effective therapeutic approach for the treatment of patients with AIS, especially in patients with large artery atherosclerosis subtype.

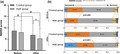

## 1 INTRODUCTION

Acute ischemic stroke (AIS) is the most common subtype of stroke with high morbidity, disability, and mortality (Feigin, 2007). Due to the aging population, the incidence of stroke and the burden that brings will increase greatly in future, especially in developing countries (Donnan, Fisher, Macleod, & Davis, 2008). The annual incidence of stroke increased by approximately 9% per year in China (Jia, Liu, & Wang, 2010). There are multiple treatment strategies for AIS patients (Cornejo‐Juarez et al., 2015; Rabinstein, 2017); however, conventional treatment is dissatisfactory. Intravenous thrombolysis with recombinant tissue type plasminogen activator (rtPA) was the most effective treatment for AIS (NBCMA, 2015). However, the treatment usually resulted in an increased risk of intracranial hemorrhage and accompanied with narrow time window and strict contraindications, which limited its clinical application (Whiteley, Slot, Fernandes, Sandercock, & Wardlaw, 2012). Currently, for a large population of patients with AIS, seeking safe and effective treatment for AIS remains the focus of global medical attention.

Human urinary kallindinogenase (HUK) is a tissue kallikrein extracted from male urine (Miao, Deng, Zhang, Xie, & Feng, 2016). It is a new drug on the market in recent years and has been approved by China Food and Drug Administration (CFDA) for the treatment of stroke. Considerable researches have confirmed that HUK could increase the blood flow and oxygen supply, improve cerebral blood circulation, and promote angiogenesis and cerebral perfusion in ischemic regions, and thus improving neurological function (Han et al., 2015; Li, Chen, et al., 2015). Besides, the safety and effectiveness of HUK for stroke patients have been verified by phase II/III clinical trials (Deyun, 2005). Nevertheless, the effective rate of HUK was approximate 80% (Li, Zha, et al., 2015), indicating the HUK was still ineffective for some patients. Previously, Li, Chen, et al. (2015) have proposed that the clinical efficacy of HUK on patients with different TOAST (The Trial of Org 10172 in Acute Stroke Treatment) type AIS was different. Thus, we hypothesized that the personalized therapy options with HUK are likely to be more appropriate for AIS patients.

Previously, Gao, Wang, Xu, Li, and Wang (2011) have provided an innovative classification system for stroke, Chinese ischemic stroke subclassification (CISS), which offered more detailed information on the pathophysiology of stroke. Therefore, based on this classification system, we hoped to find a subtype that was more suitable for HUK therapy by evaluating the effects of different subtypes on clinical efficacy. Therefore, we evaluated the effectiveness of HUK and analyzed risk factors of clinical efficacy. Furthermore, according to CISS, the effects of different subtypes on the clinical efficacy of HUK were evaluated, aiming to explore the optimal therapy strategies of HUK in the treatment of AIS.

## 2 MATERIALS AND METHODS

### 2.1 Patients

This is a retrospective, single‐center study. A total of 266 patients with AIS treated at the First Affiliated Hospital of Shantou University Medical College from September 2015 to September 2017 were enrolled in this study. The study was conducted in compliance with the Helsinki Declaration and approved by the Ethics Committee of the First Affiliated Hospital of Shantou University Medical College. Written informed consent was obtained from each subject.

Inclusion criteria were as follows: (a) Patients were diagnosed with AIS, and aged 18–90 years; (b) patients with an onset of AIS ranging from 4.5 to 72 hr; and (c) patients with the first onset or with a history of ischemic stroke but National Institute of Health Stroke Scale (NIHSS) score <4; (d) patients without bleeding disorder within the last 1 month. Exclusion criteria were as follows: (a) Patients were diagnosed with transient ischemic attack; (b) patients with obstructive cavity but without any objective signs; (c) patients with previous history of ischemic stroke, but with neurological dysfunction and NIHSS ≥ 4; (d) patients with severe liver, renal, and cardiac insufficiency, previous cerebral hemorrhage or other brain diseases; and (e) patients who are taking antihypertensive drugs (angiotensin‐inhibiting enzymes).

### 2.2 Treatment and grouping

According to the treatment program, the patients with AIS were divided into two groups: HUK group (*n* = 132) and control group (*n* = 134). All patients received conventional therapies with intravenous infusion Xuesaitong (400 mg once daily; Harbin Zhenbao Pharmaceutical Co., LTD.) and edaravone (30 mg twice daily; Yangzhou Pharmaceutical Co. LTD.) for 14 days. On the basis of conventional therapies, patients in HUK group were treated with HUK once daily (0.15 PNA unit; TECHPOOL BIO‐PHARMA Co. LTD.) for 14 days. Besides, basic treatments were performed among patients in both groups according to disease condition, including the antiplatelet therapy with aspirin (100 mg/day), lipid regulation (20 mg atorvastatin, once daily), neurotrophic treatment, and regulation of blood pressure with 0.15 g irbesartan once daily at appropriate.

According to CISS classification, the AIS patients were divided into five subtypes as follows (Gao et al., 2011): (a) large artery atherosclerosis (LAA); (b) cardiogenic stroke (CS); (c) penetrating artery disease (PAD); (d) other etiology (OE); (e) undetermined etiology (UE).

### 2.3 Outcome and clinical assessment

National Institute of Health Stroke Scale score was performed to evaluate the neurological function and clinical efficacy. NIHSS scores before and after treatment among patients in two groups were recorded. According to the improvement degree of clinical NIHSS scores, clinical efficacy was divided into six categories including: (a) basic recovery (NIHSS score decreased by more than 90%); (b) significant improvement (NIHSS score decreased by 46%–89%); improvement (NIHSS score decreased by 18%–45%); (c) no‐change (NIHSS score decreased 0%–18%, or increased <18%); (d) deterioration (NIHSS score increased more than 18%); and (e) death. The basic recovery, significant improvement, and improvement were regarded as effectivity. The no‐change, deterioration, and death were regarded as inefficiency (Qingtang, 1996; Li, Zha, et al., 2015).

For analysis, the level of homocysteine was defined as a categorical variables. Due to Chinese adults who do not eat food fortified with folic acid, the optimal threshold of homocysteine level (20 μmol/L) was determined according to the previous report (Refsum et al., 2004).

### 2.4 Statistical analysis

Statistical analysis was performed with SPSS statistics 17.0 (IBM). Mean ± standard deviation (*SD*) was used to represent the continuous variables. Qualitative data were described by number or percentage. The normality of the data distribution was tested with Kolmogorov–Smirnov test. Comparison in two groups was performed with Student's *t* test or Pearson's Chi‐square test. Logistic regression was used to identify the independent risk factors. Differences were considered statistically significant when *p* < .05.

## 3 RESULTS

### 3.1 Initial patient characteristics

The baseline characteristics of patients in two groups were shown in Table 1. Of the 266 patients with AIS enrolled in this retrospective study, 134 patients (53 females and 81 males) were treated with conventional therapies, and another 132 patients (43 females and 89 males) were treated with HUK on the basis of conventional therapies. The average age was 66.13 ± 11.08 years in HUK group and 67.40 ± 7.14 years in the control group. No significant difference of patients' characteristics (age, gender, medical history, laboratory values, NIHSS score, etc.) at admission was found in two groups (*p* > .05).

**Table 1:** Baseline characteristics of the participants

Characteristic	HUK group (*n* = 132)	Control group (*n* = 134)	*p* value
Age (years)	66.13 ± 11.08	67.40 ± 7.14	.27
Sex (female/male, %)	43 (32.6)/89 (67.4)	53 (39.5)/81 (60.5)	.24
History—no. (%)			
Smoking	37 (28.0)	34 (25.4)	.62
Drinking	11 (8.3)	16 (12.0)	.33
Hypertension	96 (72.7)	86 (64.2)	.13
Diabetes mellitus	51 (38.6)	54 (40.3)	.78
Atrial fibrillation	12 (9.1)	10 (7.5)	.63
Carotid artery stenosis	80 (60.6)	81 (60.5)	.98
Hemoglobin (g/L)	130.0 ± 17.69	129.0 ± 15.91	.64
Platelet (10^12^/L)	243.4 ± 101.5	234.6 ± 78.71	.43
Blood lipids			
TC	5.29 ± 1.43	5.20 ± 1.54	.62
TG	1.58 ± 0.89	1.70 ± 1.36	.36
LDL	3.29 ± 0.82	3.11 ± 0.84	.09
HDL	1.10 ± 0.27	1.17 ± 0.42	.12
Uric acid (μmol/L)	357.1 ± 109.3	358.9 ± 108.8	.90
Cystatin‐C (mg/L)	0.99 ± 0.37	0.95 ± 0.36	.33
Homocysteine (≤20/>20, μmol/L)	8 (6.1)/124 (93.9)	4 (3.0)/130 (97.0)	.23
Creatinine (μmol/L)	104.6 ± 32.16	107.3 ± 28.54	.48
Prothrombin time (s)	12.28 ± 1.47	12.21 ± 1.54	.69
CISS subtypes			
LAA	74 (56.1)	72 (53.7)	.98
PAD	33 (25.0)	35 (26.1)	
CS	12 (9.1)	11 (8.2)	
UE	7 (5.3)	8 (6.0)	
OE	6 (4.5)	8 (6.0)	
NIHSS score at admission	7.98 ± 5.27	8.65 ± 5.16	.30

Abbreviations: CISS, Chinese ischemic stroke subclassification; CS, cardiogenic stroke; HDL‐C, high‐density lipoprotein cholesterol; LAA, large artery atherosclerosis; LDL‐C, low‐density lipoprotein cholesterol; NIHSS, National Institute of Health Stroke Scale, ranged from 0, indicating normal functioning, to 42, indicating most severe impairment; OE, other etiology; PAD, penetrating artery disease; TC, total cholesterol; TG, triglycerides; UE, undetermined etiology.

According to the CISS classification, there were 74 (56.1%) cases of LAA subtype, 33 (25.0%) cases of PDA, 12 (9.1%) cases of CS, 7 (5.3%) cases of UE and 6 (4.5%) cases of OE in HUK group, and 72 (53.7%) cases of LAA subtype, 35 (26.1%) casesof PDA, 11 (8.2%) cases of CS, 8 (6.0%) cases of UE, and 8 (6.0%) cases of OE in the control group. The distribution of CISS classification was not different between the two groups (*p* = .98).

### 3.2 NIHSS scores and efficacy evaluation before and after treatment

**Figure 1:**
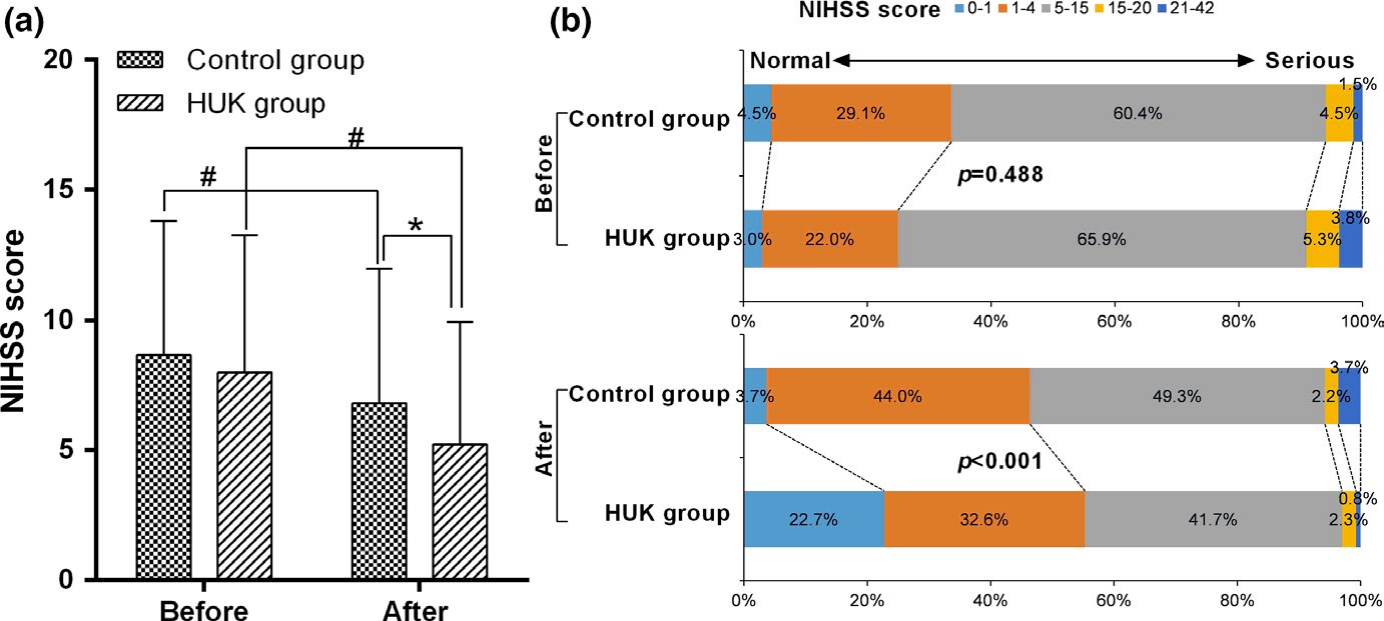
Neurological function evaluated by NIHSS score. (a) The changes in NIHSS score before and after treatment. (b) The distribution of the NIHSS scores in two groups after treatment. *p < .05 versus control group; ^#^ *p* < .05 versus at admission. HUK, Human urinary kallindinogenase; NIHSS, National Institute of Health Stroke Scale

At baseline, no significant difference was found in NIHSS scores between two groups (*p* = .30). After receiving the specified treatment for 14 days, NIHSS scores in two groups were all significantly reduced compared with that before treatment (*p* < .001, Figure 1a). Besides, the NIHSS score of HUK group was significant lower than that of control group, indicating that HUK has significant effect on improving the neurological function of ASI patients (*p* = .009). In addition, the distribution of the NIHSS scores changed before and after treatment (Figure 1b). At baseline, 4.5% of patient who had no stroke symptoms (NIHSS score <1) were found in control group, and 3.0% of the patients in HUK group. Additionally, 1.5% and 3.8% of the patients had a severe stroke (NIHSS score: 21–42) were found in the control group and HUK group, respectively. No significant difference was observed in the distribution of the NIHSS scores before treatment (*p* = .488). After treatment, 22.7% and 3.7% of the patients without stroke symptoms (NIHSS score <1) were found in the HUK group and control group, respectively. Severe strokes (NIHSS score: 21–42) were found in 0.8% of patients in the HUK group and 3.7% of the patients in the control group (*p* < .001), indicating a good outcome in HUK group.

After treatment, basic recovery was found in 13 patients, significant improvement 40, improvement 42, no‐change 30, and deterioration 7 in HUK group, the effectiveness rate was 71.2% (95 patients). In the control group, basic recovery was found in one patient, significant improvement 31, improvement 40, no‐change 54, and deterioration 8, the effectiveness rate was 53.7% (72 patients). Pearson's Chi‐square result showed that the total effectiveness rate of HUK group was significant higher than that of control group (*p* = .003).

### 3.3 NIHSS scores and efficacy evaluation of different subtypes

**Figure 2:**
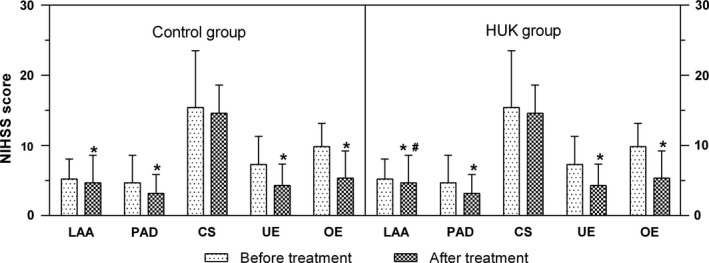
The changes of NIHSS score in five subtypes before and after treatment. CS, cardiogenic stroke; HUK, human urinary kallindinogenase; LAA, large artery atherosclerosis; OE, other etiology; PAD, penetrating artery disease; UE, undetermined etiology

With regard to the five subtypes of CISS classification, no significant difference was found between the subtypes of the two groups at baseline (*p* > .05, Figure 2). After treatment, a significant reduction of NIHSS score was measured for every subtype except CS subtype, in both two groups (LAA, PAD, UE, OE: *p* < .05; CS: *p* > .05). Besides, after treatment, the NIHSS score of patients with LAA subtype in HUK group was found to be significant lower than that in control group (*p* = .005, Figure 2).

The clinical efficacy for patients with different subtypes was showed in Table 2. The effectiveness rate of LAA subtype showed a statistically significant difference between two groups (*p* = .004, Table 2), while the other subtypes showed no significant difference between two groups (*p* > .05, Table 2).

**Table 2:** Comparisons of clinical efficacy between two groups according to the CISS subtypes after treatment

Grouping	Effectivity/Ineffectiveness				
	LAA	PAD	CS	UE	OE
HUK group (*n* = 132)	54 (73.0%)/20 (27.0%)	25 (75.8%)/8 (24.2%)	3 (25.0%)/9 (75.0%)	7 (100.0%)/0 (0.0%)	6 (100.0%)/0 (0.0%)
Control group (*n* = 134)	36 (50.0%)/36 (50.0%)	25 (57.1%)/15 (42.9%)	3 (27.3%)/8 (72.7%)	8 (100.0%)/0 (0.0%)	5 (62.5%)/3 (37.5%)
*p* value	.004	.105	>.999	—	0.209

Abbreviations: CS, cardiogenic stroke; LAA, large artery atherosclerosis; OE, other etiology; PAD, penetrating artery disease; UE, undetermined etiology.

### 3.4 Risk factors analysis

Table 3 provides the overall odds ratios (OR) for individual risk factors performed by univariate logistic regression analysis. Results showed that the use of HUK, atrial fibrillation (AF), homocysteine, CS subtype, and NIHSS score were significantly related to the clinical efficacy (*p* < .05, Table 3). The use of HUK was associated with a better clinical efficacy of HUK (OR = 2.21, 95% CI: 1.33–3.68, *p* = .002). In addition, atrial fibrillation, high level of homocysteine, CS subtype, and high NIHSS score were associated with a worse clinical efficacy (AF: OR = 0.19, 95% CI: 0.07–0.51, *p* = .001; homocysteine: OR = 0.18, 95% CI: 0.05–0.69, *p* = .01; CS subtype: OR = 0.22, 95% CI: 0.08–0.59, *p* = .003; NIHSS: OR = 0.94, 95% CI: 0.90–0.99, *p* = .02).

**Table 3:** The risk factors for clinical efficacy in univariate logistic regression analysis

Characteristic	OR	95% CI	*p* value
Age	0.986	0.960–1.013	.315
Sex	0.950	0.566–1.596	.848
HUK	2.211	1.328–3.679	.002
Smoking	0.748	0.430–1.304	.306
Drinking	0.715	0.320–1.598	.414
Hypertension	0.981	0.574–1.675	.943
Diabetes mellitus	0.629	0.379–1.045	.073
Atrial fibrillation	0.193	0.073–0.513	.001
Carotid artery stenosis	0.995	0.598–1.654	.984
Hemoglobin	1.009	0.994–1.024	.242
Platelet	0.998	0.996–1.001	.246
TC	0.994	0.840–1.175	.941
TG	0.859	0.690–1.070	.176
LDL	0.766	0.567–1.035	.082
HDL	1.447	0.677–3.095	.340
Uric acid	1.000	0.998–1.002	.875
Cystatin‐C	0.586	0.298–1.154	.122
Homocysteine (≤20 μmol/L)	1.000	1.000–1.000	1.000
Homocysteine (>20 μmol/L)	0.183	0.048–0.693	.012
Creatinine	0.999	0.991–1.008	.896
Prothrombin time	0.989	0.838–1.167	.897
NIHSS score at admission	0.944	0.900–0.990	.019
CISS subtypes			
LAA	1.000	1.000–1.000	1.000
PAD	1.217	0.666–2.225	.523
CS	0.220	0.082–0.590	.003
OE	2.281	0.610–8.536	.221

Abbreviations: CISS, Chinese ischemic stroke subclassification; CS, cardiogenic stroke; HDL‐C, high‐density lipoprotein cholesterol; HUK, human urinary kallindinogenase; LAA, large artery atherosclerosis; LDL‐C, low‐density lipoprotein cholesterol; NIHSS, National Institute of Health Stroke Scale; OE, other etiology; PAD, penetrating artery disease; TC, total cholesterol; TG, triglycerides; UE, undetermined etiology.

After multivariate analysis, the absence of HUK, homocysteine, and CS subtype were found to be the risk factors for the clinical efficacy of HUK (HUK: OR = 2.75, 95% CI: 1.58–3.82 *p* = .01; homocysteine: OR = 0.15, 95% CI: 0.04–0.61, *p* = .01; CS subtype: OR = 0.18, 95% CI: 0.06–0.51, *p* = .001; Table 4), indicating that the use of HUK and the lower level of homocysteine were protective for the clinical efficacy of HUK.

**Table 4:** The risk factors for clinical efficacy in multivariate logistic regression analysis

Characteristic	OR	95% CI	*p* value
HUK	2.211	1.328–3.679	.002
Homocysteine (≤20 μmol/L)	1.000	1.000–1.000	1.000
Homocysteine (>20 μmol/L)	0.153	0.038–0.612	.008
CISS subtypes			
LAA	1.000	1.000–1.000	1.000
PAD	1.241	0.662–2.328	.501
CS	0.190	0.068–0.530	.002
OE	2.322	0.602–8.956	.221

Abbreviations: CISS, Chinese ischemic stroke subclassification; CS, cardiogenic stroke; HUK, human urinary kallindinogenase; LAA, large artery atherosclerosis; OE, other etiology; PAD, penetrating artery disease; UE, undetermined etiology.

## 4 DISCUSSION

In China, HUK is recommended to be used for improving cerebral blood circulation in patients with AIS (NBCMA, 2015). An economic evaluation study has revealed that HUK is a cost‐saving therapy for the management of AIS (Lin, Rao, Zhang, & Xuan, 2018). Thus, HUK is very likely to become a promising treatment medicine for AIS in a Chinese setting. However, the therapeutic efficacy of HUK was limited in some patients with AIS. Thus, the optimal therapeutic regimen for patients with different subtypes AIS is urgently needed to be explored. To our knowledge, this is the first assessment of the clinical efficacy of HUK therapy for AIS patients according to the CISS classification.

We confirmed that HUK significantly improved the neurological function of AIS patients with total effectiveness rate of 71.2%. These results are consistent with previous research reports (Song et al., 2018; Wei et al., 2018). A systemic review has also demonstrated that 2,117 patients (22 trials) benefited from HUK treatment and the efficacy rate was 87% in China (Zhang, Tao, Liu, & Wang, 2012). Several hypotheses have been proposed to explain the mechanism of HUK on AIS (Chen et al., 2010; Han et al., 2015; Li, Chen, et al., 2015). HUK is a tissue kallikrein, could cleave low molecular weight kininogen to vasodilator kinin peptide (Emanuelia & Madeddu, 2003). Then, it triggers a series of biological effects by activating bradykinin B1 and B2 receptors through the KKS system (Campbell, 2001; Emanuelia & Madeddu, 2003). Another explanation was that HUK could enhance angiogenesis in the subventricular zone and increases capillary density in cerebral peri‐infarct area, which in turn improves collateral circulation (Han et al., 2015). Besides, Song et al. have reported that HUK improved symptoms of neurological deficiency by enhancing remodeling of long‐term cortical motor function in patients with AIS (Song, Han, & Liu, 2012). Although multiple works have focused on the mechanism of HUK, conclusion was not definitively made. Further studies on a large scale are still needed to verify the effect of HUK in clinical practice.

The highlight of this study was the finding of better clinical efficacy of HUK therapy in LAA subtype patients, indicating that the HUK therapy is more appropriate for patients with LAA subtype than the other subtypes. A previous study has reported the clinical efficacy of HUK in the treatment of AIS according to TOAST classification (Li, Zha, et al., 2015). They came to a similar conclusion with us. In our study, CISS was used for the classification, which was an improved and more rational way of stroke subtyping that takes into consideration of etiological and pathophysiological information (Chen et al., 2012). On the basis of CISS, LAA is the most common cause of ischemic stroke (Gao et al., 2011). Patients with LAA subtype were prone to form atherosclerotic thrombosis and thus may result in arterial stenosis or occlusion. Additionally, most LAA subtype patients typically presented with chronic onset and accompanied by the opening of collateral circulation. Therefore, the use of HUK in the acute stage of ischemic stroke could promote angiogenesis, improve collateral circulation, and eventually significantly improve its clinical treatment. However, it should be noted that patients with CS subtype did not benefit from HUK therapy. The reason may be that the lesions of patients with CS subtype mostly involved main artery (Tan et al., 2016). It is difficult to establish a rapid collateral circulation in acute phase, leading to the absence of effective collateral circulation even with the use of HUK. Based on findings of this study, we assumed that HUK treatment may not be appropriate for patients with CS subtype. Thus, we recommended that clinicians should diagnose the subtype classification of patients with stroke, so as to perform the personalized treatment for different patients. However, further randomized studies with a larger number of samples are still needed to verify these findings.

We also analyzed the risk factors of HUK clinical efficacy and found that the use of HUK and the lower level of homocysteine were protective factors for the clinical efficacy of HUK. There is no doubt that patients with AIS would be benefited from HUK therapy, which has been confirmed by considerable clinical trials (Ni et al., 2017; Zhang et al., 2012). The relationship between homocysteine and HUK clinical efficacy is worthy of attention. Some previous reports have demonstrated that elevated homocysteine was a risk factor for ischemic stroke (Homocysteine Studies Collaboration, 2002). That may explain our finding that a lower level of homocysteine was protective for the clinical efficacy of HUK.

Although some important discoveries were revealed by our study, there are still several limitations. First, the relatively small sample size of patients in this study may lead to a bias of our results. Secondly, our study had its inherent limitations of single‐center, nonrandomized, and retrospective design, and thus further studies are still warranted. Finally, long‐term follow‐up research is needed because the limited follow‐up may miss some later recurrences.

In conclusion, HUK could significantly improve the neurological function of AIS patients. According to the CISS classification, HUK therapy is more appropriate for patients with LAA subtype than the other subtypes, while had no efficacy for CS subtype. The absence of HUK, elevated homocysteine, and CS subtype were risk factor for the clinical efficacy of HUK. However, further studies with a larger number of samples and long‐term follow‐up are warranted to determine the personalized treatment of HUK.

## CONFLICT OF INTEREST

The authors declare that they have no conflict of interest.

## Data Availability

The data that support the findings of this study are available from the corresponding author upon reasonable request.
